# Adapting the Israeli national health insurance law to the 21^st^ century– a report from the 19^th^ Dead Sea Conference

**DOI:** 10.1186/s13584-020-00432-y

**Published:** 2021-01-05

**Authors:** Yoel Angel, Adi Niv-Yagoda, Ronni Gamzu

**Affiliations:** 1 Tel Aviv Sourasky Medical Center, Weizmann 6, 6423906 Tel Aviv, Israel; 2 Sackler Faculty of Medicine, Tel Aviv University, Tel Aviv, Israel; 3 Coller School of Management, Tel Aviv University, Tel Aviv, Israel; 4 School of Health Systems Management, Netanya Academic College, Netanya, Israel

**Keywords:** Health policy, National Health Insurance, Conference proceedings

## Abstract

Passage of the National Health Insurance Law (NHIL) in 1995 marked a turning point in the history of the Israeli healthcare system, ensuring sustainable, high-quality medical care to all eligible Israeli residents. Over 100 amendments have been made to the law over the years, yet additional adaptations are required to ensure the law’s relevance in years to come. In honor of the 25th anniversary of the passage of the law, the 19th annual Dead Sea Conference brought together prominent figures in the Israeli healthcare system for a discussion on “25 Years to the NHIL: Suggested Changes and Adaptations”. Key topics discussed in the conference were regulatory aspects related to the healthcare system, administration of medical services, and financial aspects pertinent to the NHIL. The following meeting report summarizes the insights and recommendations from this conference.

## Background

In June 1994, the Israeli parliament passed the National Health Insurance Law (NHIL), a turning point in the history of the Israeli healthcare system. After decades of repeated financial crises, poor quality of care, and low public satisfaction, the system underwent an overhaul, with the goal of ensuring the sustainability of the Israeli healthcare system while adhering to the declared principles of “justice, equality and mutual assistance”. For the first time, a mandatory, government-funded, national health basket was defined, to be provided by the four existing health insurance organizations, known as Sick Funds, while certain services remained under direct responsibility of the Ministry of Health (MoH). Furthermore, an obligatory health tax was created, earmarked to fund these health services, thus ensuring the system’s sustainability. The mechanisms for joining a Sick Fund were formalized, putting an end to the problematic dependency between political party affiliation or workers’ union membership and eligibility for healthcare services. This law also put an end to patient selection by the Sick Funds, which contributed to “cream skimming” and inequal expenditure between the four funds. Government funding for the Sick Funds was allocated based on pre-determined criteria that take into account the number of members in each fund, as well as their demographic characteristics. These, along with other reforms included in the law, brought about the single greatest change in the history of the Israeli healthcare system [1–3]. Legislative efforts continued over the years, and over 100 amendments have been made to the NHIL since its initial passage, some minor and technical in nature, while others brought about fundamental change [4]. The core of the law, however, remained unchanged. Israel takes pride in its healthcare system, which has gained international acclaim for its strong outcomes and low national spending [5]. Much of this achievement can be attributed to the NHIL and the reforms that came with it. At the 25th anniversary of the passage of the law, the National Institute of Health Policy’s annual Dead Sea Conference (see Table 1) was dedicated to examination of the adequacy of the law for the current reality and possible adaptions to be made to it, in order to ensure the continued relevance of the law for the near and distant future.

**Table 1:** About the dead sea conference

The Dead Sea Conference has been held annually since the year 2000. The conference is organized by the National Institute of Health Policy Research, a statutory non-profit organization devoted to the promotion of health policy research in Israel (and also the publisher of this journal). The conference is designed as a workshop, bringing together over one hundred leaders and key stakeholders from the entire Israeli healthcare ecosystem, including the Ministry of Health, national Sick Funds, government hospitals, academic institutions, and more. The conference serves as a platform where these experts can shed their institutional obligations and freely discuss prominent issues on the healthcare system’s agenda. The conference is the climax of several months of diligent preparation by a multidisciplinary organizing committee, based on the specific theme selected for that year’s event. Previous conventions have addressed issues related to the structure of the healthcare system, such as hospital incorporation, private health services, and more, as well as aspects related to administration of medical care such as medical malpractice, technology in medicine, and quality metrics, to name only a few of the many topics discussed. The conference’s content and recommendations are discussed in detail and distributed in an official publication by the National Institute of Health Policy Research (in Hebrew). While the recommendations have no binding status, they are often used by different government and academic institutions in Israel and serve as the foundation for legislation and reform efforts within the Israeli healthcare system.	

## Discussions, conclusions, and recommendations

The conference was largely organized around three parallel group discussions: (1) regulation of the healthcare system; (2) aspects relating to the administration of medical services; and (3) financial aspects pertinent to the National Health Insurance Legislation. Sessions were led by chairpersons based on material that was prepared and distributed before the conference. Several plenary sessions were also held, including (1) a review of the theoretical basis for legislation processes and the practical barriers often encountered; (2) a first-hand historical review of the NHIL legislation process; (3) a review of the degree to which recommendations from the previous ten Dead Sea Conferences have been implemented; and (4) an analysis of the Israeli healthcare system from a socio-economic standpoint, with estimations for required additional resources, suggested allocation of these resources within the system, and possible funding sources. Key recommendations from the three tracks were presented in a concluding plenary session and are summarized in Table 2. The following is a review of these recommendations.

**Table 2:** Overview of the key recommendations from the convention

Regulation of the healthcare system	Improvement of the oversight of public hospitals by explicitly recognizing them in the NHIL and expanding the “regulatory arsenal” available to the MoH. Reduction of the overall regulatory burden by promoting discussions between the MoH and regulated entities on planned regulatory directives and by deprecation of obsolete or irrelevant ones. Improvement of the flow of information between the MoH and regulated entities by tunneling requests through a single point of contact in each organization and by creating automatic interfaces where possible.
Administration of medical services	Increasing accessibility to care by routinely measuring wait times, making the results public and enforcing improvement in cases where market forces fail to do so. Promotion of transparency by informing the public about contracted service providers available to choose from. Improvement of continuity and quality of care by transferring most of the services still provided directly by the MoH (the “Third Addition” to the NHIL) to the responsibility of the Sick Funds. Expansion of preventive medicine services by transferring them to the responsibility of the Sick Funds and prioritizing additional services for inclusion in the mandatory Health Basket.
financial aspects of the healthcare system	Adapting the healthcare budget to realistic needs by periodic re-evaluation of budgetary indices and of the capitation formula and by switching to five-year budgetary agreements. Reduction of rising healthcare costs attributable to the private health sector by improving the regulation of private health insurance and by considering reimbursement of the public sector by the private sector. Exploration of additional mechanisms to limit the rise in physicians’ wages. Curbing the spending on medication and medical technology by exploring novel initiatives such as pay-for-performance acquisition models or international collaboration for procurement of medications.

*NHIL* National Health Insurance Law, *MoH* Ministry of Health

### Group 1: regulation of the healthcare system

Public healthcare systems, perhaps more so than other public services, require effective regulation to address inherent market failures such as supply-induced demand, information asymmetry between provider and consumer, financial incentives not necessarily in line with public interest, and more. The purpose of regulation is to promote efficient, fair, and just allocation of public resources to ensure maximal benefit to the population. Albeit, excessive regulation may also be detrimental, as it may consume resources from the regulator as well as the regulated bodies disproportionately to the benefit gained.

There was a consensus among the participants in this group that effective and focused regulation is key to the proper function of the healthcare system as a whole, and is vital for ensuring that public interest and the goals of the NHIL are met. However, such regulation must ensure the autonomy of the supervised entities and avoid excessive regulatory overhead. Several topics related to regulation were discussed:

#### Oversight of hospitals

The NHIL does not specifically refer to hospitals (whether government, Sick Fund, or privately owned), as these are implicitly regarded as service providers for the Sick Funds. Hospitals are subject to oversight and regulation by several divisions in the MoH and other government ministries, without a clear, single point of contact. Furthermore, the current set of laws defines inadequate sanctions and incentives to ensure compliance with regulatory directives. For example, while the MoH has the right to close a hospital for non-compliance with directives, such an extreme measure is not feasible given the already low number of hospital beds per capita [5] in Israel. Group members therefore drafted the following recommendations with a high level of consensus:

A The NHIL should be amended to explicitly recognize hospitals as a separate entity in the Israeli healthcare system, and to define applicable regulation and oversight mechanisms for them, including mandatory reporting and transfer of information to the MoH, similar to what is required of other entities already recognized by the NHIL.
B A database of various aspects of hospital activities should be created as a platform for effective and efficient oversight. The data collected should include waiting times, available services, number of procedures performed, etc.
C Applicable legislation should be amended to expand the range of tools available to regulators, to ensure compliance with regulatory directives.

Further suggestions that did not reach a consensus but warrant further discussion included improving the corporate governance of hospitals (for example, by requiring boards of directors); increasing oversight of Health Corporations (entities owned by government hospitals for the purpose of using hospital infrastructure to provide additional, for-profit services); and resolving the MoH’s inherent conflict of interest as both owner of government hospitals and regulator of all hospitals.

#### Oversight of sick funds

There was a broad agreement that there is room for improvement in the current regulatory ecosystem and that the available regulatory tools must be revised in order to be more effective. However, some participants argued that the current regulatory system is ineffective because it imposes an excess financial, managerial, and labor burden on the regulated entities and inhibits autonomous growth, while others argued that it is the compliance with regulatory directives that should be improved. There was also a consensus that while personal accountability of high-ranking officials is warranted, imposing financial sanctions on these officials to enforce accountability is not. All participants agreed that promotion of mutual trust between the MoH and the Sick Funds is the foundation for future progress in this field. The group made the following recommendations:

A An open channel of communication should be formed between the regulator and the regulated entities, to facilitate an exchange of comments and discussions about planned rules and regulations before they come into effect.
B Periodic evaluation of existing regulatory directives should be performed to identify obsolete and inadequate directives that do not contribute to improving the quality of services, thus reducing the regulatory burden.

The group also recommended further discussion on the proper mechanisms necessary to ensure the personal accountability of executives in the healthcare system.

#### Information as a foundation for regulation

It was agreed that effective regulation requires an accurate, up-to-date and comprehensive database. However, there was a lack of consensus about the nature of the information to be collected by the regulator, as well as the mechanisms for the transfer of information from the regulated entities to the regulator. Specifically, numerous requests for information received from multiple sub-units of the MoH impose a high financial and labor burden on the Sick Funds, while the cost-to-benefit ratio of these requests is unclear. Group members were therefore in favor of holding further meetings to define in detail precisely which data is required by the MoH to fulfill its regulatory role, in order to minimize requests for information that is either irrelevant or outside the scope of the MoH’s role as a regulator. The group made the following recommendations:

A The MoH should recognize the financial implications and the human resources required to enable the regulated entities to respond to its requests for information.
B Requests for information by the MoH should be channeled via a single office within the MoH that will also be responsible for refining and prioritizing these requests, with respect to the regulated entities’ capacity to respond.
C Efforts should be made to develop a shared platform for automatic transfer of such information, while minimizing the manual labor, costs, and timelines required for transfer of such information in the future.
D Further discussion is warranted to examine the possibility of drafting a bilateral Service Level Agreement (SLA) for response times to requests between the MoH and the regulated entities, and vice versa

### Group 2: administration of medical services

#### Availability of medical services

Unlike many other aspects of the Israeli healthcare system that are directly addressed by the NHIL, regulation of the administration of medical services remains vague. The law states that medical services shall be given “…according to medical discretion, with reasonable quality, within a reasonable timeframe and at a reasonable distance from the patient’s residence, subject to the financial resources available to the Sick Fund” [6]. This language leaves much room for the Sick Funds’ judgement, and anticipates that market forces and competition between the Sick Funds would promote optimal quality of services with the available resources. While the participants in this discussion agreed that the quality of medical care in Israel is adequate and that geographic proximity is becoming less relevant in the era of telemedicine, the availability of medical care (i.e., waiting times) remains the major weakness of the public healthcare system, especially given the continuous shortage in resources. Indeed, waiting times for some non-urgent procedures have been reported to be as long as 5 months [7]. Several recommendations were made to address this issue:

A Efforts should be made to improve the span and methodology of measuring waiting times and to make the results public, as an incentive for the Sick Funds to improve their services in this area. The data that are measured and reported should consider different population segments (elderly, pediatric, etc.), providers (hospitals and outpatient care), and the possibility of excessive resource allocation for the parameters being measured (“the multitasker’s problem”).
B In instances where a market failure creates an ongoing and excessive delay in services, the regulator should set specific limits on waiting times, while addressing any root causes of the problem that are under the MoH’ purview.
C In specific cases such as remote and sparsely populated areas, the regulator should consider developing a mechanism for pooling resources between different Sick Funds to form an ad-hoc, combined entity that can benefit from an economy of scale.

#### Choice of service providers

The Sick Funds, acting as representatives of their insurees, periodically negotiate agreements with certain service providers, thus optimizing public spending. As a result, referral of patients to these providers is often motivated more by the Sick Fund’s financial interests rather than the patient’s preferences, without transparency towards the public. It was therefore agreed that:

A Provider agreements play an essential role in curbing public spending and therefore should continue to exist.
B The public should be explicitly notified of the providers available, according to the choice arrangements in effect for each Sick Fund.
C Patients should be allowed to engage providers outside the network of available providers when waiting times exceed a pre-determined threshold. This will benefit the patients and incentivize Sick Funds to ensure adequate provider agreements.

#### Transfer of services from the MoH to the sick funds

For historical reasons, some services, such as rehabilitation devices, preventive services, and geriatric long-term hospitalization, are provided directly by the MoH. The first version of the NHIL implicitly states that this is an interim arrangement that is destined to be changed – however several attempts over the years to transfer these services to be under the purview of the Sick Funds have failed for various reasons. This, of course, preserved an anomaly in which the MoH acts as a provider rather than a regulator. In order to fix this anomaly, the following guidelines were recommended:

A The responsibility for provision of services should be transferred to the Sick Funds wherever this improves continuity of care and/or wherever the MoH serves only as a funding source and does not handle actual administration of care.
B A joint team of representatives of the MoH and the Sick Funds should be formed to make specific recommendations as to which services should be transferred and when.
C Family health clinics (formerly, well-baby clinics) are considered one of the Israeli healthcare system’s greatest accomplishments [8]. No consensus was reached regarding whether these clinics should remain within the purview of the MoH or become the Sick Funds’ responsibility, as there are advantages to each of the operating models.

#### Preventive medicine

As mentioned, administration of preventive medicine services (such as vaccinations and health education) is also the direct responsibility of the MoH. While the MoH does have specific advantages in some areas (such as schoolchildren’s health, which requires intense state-level coordination with other government entities), in most cases the Sick Funds have a much stronger interest in promoting preventive medicine services (to reduce potential future spending). It was therefore agreed that:

A In general, most preventive medicine services should become the responsibility of the Sick Funds, and any necessary changes to the NHIL should be made to allow this to happen.
B As the medical technologies added to the health basket each year are skewed towards therapeutic interventions, a mechanism (such as a separate fund or mandatory fixed percentage of each addition) should be put in place to ensure that novel preventive medicine technologies are routinely added to the annual health basket.

### Group 3: financial aspects of the National Health Insurance Legislation

The NHIL played a pivotal role in defining and ensuring adequate funding for healthcare services included in the mandatory “health basket”, while simultaneously creating checks and balances on national spending on health. Indeed, since its legislation in 1995, national spending on health has increased by only 7% (from 6.9 to 7.4% of the GDP), compared to the 23% OECD average [9]. However, a series of changes over the last 25 years in the economic environment may jeopardize the continued effectiveness of the mechanisms that are at the base of the NHIL. Specifically, the rising cost of medical technologies and state-of-the-art medications, and the continued increase in doctors’ wages due (in part) to the expansion of the private health sector in Israel, all put greater demands on the limited public health budget. At the same time this budget does not adequately reflect changes in the population’s size, demographics, and medical complexities. Today, the health basket budget is allocated to the Sick Funds (“slicing of the pie”) according to a capitation formula that includes only age, gender and area of residence, in addition to compensation for a small number of severe diseases on a per-capita basis. Adjustment of the health basket budget to the changing landscape (“the size of the pie”) is achieved by linking it to two key indices mentioned in the NHIL– demographics and production costs. A third mechanism that has become standard practice is a periodic addition to the base of the budget to compensate for new technologies [3]. However, the formula for the aforementioned indices is only seldom adjusted, and the periodic addition is allocated annually from the government’s budget, so that adjustments to the health basket budget depend on yearly negotiations between the MoH and the Ministry of Finance (MoF) instead of being automatic. Some of these adjustments are made to the base of the budget while others are a one-time event. Due to the continuous under-funding of the Sick Funds, periodic government subsidies to the Sick Funds, or “stabilization grants”, have become routine. Often, the MoH and MoF condition these subsidies on achieving set goals or on unrelated organizational adaptations being made by the Sick Funds.

With the common goal of ensuring the financial stability of the Israeli healthcare system for years to come, especially in light of the expected demographic changes in the Israeli population [10], group members made several recommendations in three key areas:

#### Health basket funding

While some participants believed that budget indices need to be automatically adjusted, others believe that it is necessary to leave this to the judgement of the MoF, while also considering other national priorities. There was a consensus that it is imperative to update the different indices. Group members agreed on the following recommendations:

A A database to evaluate the different patient-level parameters influencing healthcare spending should be developed, to continuously improve the capitation formula.
B A permanent committee should be formed to evaluate the composition of the two budget indices as well as the capitation formula, and make recommendations to the MoH and MoF regarding periodic updates of these indices.
C A reevaluation of the practice of “stabilization grants” should be performed.
D The duration of budget agreements made in the healthcare system, between the MoH and the MoF and between the MoH and the Sick Funds, should possibly be extended from 3 to 5 years, to allow for long-term budget planning.

#### Private health insurance

The private health sector in Israel has seen a significant rise in market share, with 36% of national health expenditure coming from non-public sources [5]. Private insurance in Israel is composed of voluntary health insurance offered by the Sick Funds and commercial health insurance. According to a report by the Brookdale institute in 2016, close to 85% of households owned the Sick Funds’ voluntary insurance and 57% owned commercial insurance [11]. While the former is specifically mentioned in the NHIL and is regulated by the MoH, the latter is regulated solely by the Authority for Capital Market, Insurance and Savings (an independent body within the MoF), which focuses more on fiscal aspects of insurance rather than on the effects of the private health sector on the public sector. Notably, this regulatory lacuna was addressed in the 2012 Dead Sea Conference as well [12]. It is widely agreed that the private sector has significant competitive advantages over the public sector, such as “cream skimming” for profitable procedures on low-complexity patients, and indirect subsidies by the public sector (e.g. training of medical personnel). Moreover, the significantly higher salaries in the private sector and competition over production resources (i.e. medical personnel) with the public sector has caused an “upward spiral” of spending on salaries and has had a negative effect on the financial stability of the public healthcare system.

Therefore, participants agreed that prices in the private healthcare sector require oversight. The group made the following recommendations:

A The MoH should be granted standing in the oversight of commercial health insurance.
B A permanent committee should be formed, with representation by both the Authority for Capital Market, Insurance and Savings and the MoH, to evaluate and address the cross effects of commercial and public health insurance.
C Cost restriction policies should be applied on privately-operated service providers.
D A committee should be formed to quantify the financial influences of the private health sector on the public sector, and consider instating mechanisms for the private sector to reimburse the public sector for these costs, for example by imposing a dedicated tax or allowing for subrogation by the public sector for additional costs incurred.

#### Curbing of healthcare spending

The two factors that were identified as contributing the most to the increase in healthcare spending are physician wages and spending on medication (especially personalized, state-of-the-art medications). For example, the average salary of a specialist in Family Medicine rose by 60% between 2010 and 2017 [internal MoH unpublished data]. The increase in physician wages was attributed to fierce competition between the four Sick Funds, and between the private and public healthcare sectors. Obtaining exact data and diligent oversight of physicians’ wages was found to be extremely complicated, due to the wide variety of contract types between physicians and Sick Funds (e.g. direct employment, contract with a single physician or with a clinic which also provides additional services, etc.). Expenditure to include new medications in the health basket has also risen substantially, with the average cost of a new medication in 2019 being up to seven times higher than in 2008 [unpublished MoH data]. One of the possible reasons for the increased spending is the individual, case-by-case (yet not at all rare) approval of medications and treatments by the Sick Funds’ internal exceptions committees, beyond the scope of the government-defined, obligatory health basket (which is matched by adequate resources). In light of these observations, the group made the following recommendations:

A Additional research is required to identify all the available methods used by Sick Funds to contract with physicians, and to propose mechanisms for overseeing physicians’ wages in each of these models.
B Voluntary standardization of independent (contracted) physicians’ wages by the Sick Funds, according to specialty and geographic region, should be encouraged.
C The existing mechanisms for price regulation should be expanded to include medical devices, expendables, and non-registered medications.
D The regulatory process for registering generic substitutes and biosimilar drugs should be expedited and simplified, as a means for encouraging market-wide price reductions.
E International collaboration that can help curb pharmaceutical prices should be encouraged.
F Novel, innovative models for acquisition of new medications, such as risk sharing and pay-for-performance, should be considered.
G Further research is required to evaluate the adequacy and effectiveness of the internal Sick Fund exceptions committees, while considering combining them into a single, national exceptions committee.

## Summary

The NHIL is the cornerstone of the Israeli healthcare system and is expected to remain as such for the foreseeable future. The conference highlighted issues that are key to promoting the efficiency and quality of the public medical services or that may jeopardize the long-term financial sustainability of the Israeli healthcare system. The recommendations made by the three working groups should serve as a basis for future amendments to the law.

## Data Availability

Not applicable.
